# The Microenvironment in Gliomas: Phenotypic Expressions

**DOI:** 10.3390/cancers7040896

**Published:** 2015-12-03

**Authors:** Davide Schiffer, Laura Annovazzi, Marta Mazzucco, Marta Mellai

**Affiliations:** Research Center, Policlinico di Monza Foundation, Via Pietro Micca 29, 13100 Vercelli, Italy; laura.annovazzi@cnbo.it (L.A.); marta.mazzucco91@gmail.com (M.M.); marta.mellai@cnbo.it (M.M.)

**Keywords:** stem cells, microenvironment, niches

## Abstract

The microenvironment of malignant gliomas is described according to its definition in the literature. Beside tumor cells, a series of stromal cells (microglia/macrophages, pericytes, fibroblasts, endothelial cells, normal and reactive astrocytes) represents the cell component, whereas a complex network of molecular signaling represents the functional component. Its most evident expressions are perivascular and perinecrotic niches that are believed to be the site of tumor stem cells or progenitors in the tumor. Phenotypically, both niches are not easily recognizable; here, they are described together with a critical revision of their concept. As for perinecrotic niches, an alternative interpretation is given about their origin that regards the tumor stem cells as the residue of those that populated hyperproliferating areas in which necroses develop. This is based on the concept that the stem-like is a status and not a cell type, depending on the microenvironment that regulates a conversion of tumor non-stem cells and tumor stem cells through a cell reprogramming.

With the term “tumor microenvironment” it is intended to indicate everything is active within the tumor except tumor cells. It includes, therefore, many cell types, such as endothelial cells, microglia/macrophages, reactive astrocytes, fibroblasts, pericytes, immune cells, *etc.*, [1] and the relevant factors and molecular signaling addressed to promote tumor transformation, growth, invasion, therapeutic resistance [2] and defense from host immunity [3,4].

Any regulation of tumor cells towards regression, such as necrosis, or progression, such as proliferation, invasion and angiogenesis, finds its main driver in the microenvironment that mainly expresses itself in the so-called niches [3,4,5]. As a matter of fact, in glioblastoma multiforme (GBM), niches are regarded as the crucial points where microenvironment exerts influence, since they are the sites where glioblastoma stem cells (GSCs) are believed to reside, be maintained or originate and where the signaling arising in stromal and in tumor cells converges to regulate tumor features. Niches can be perivascular (PVN) or perinecrotic (PNN). The former have been conceived as simply represented by endothelial cells associated with Nestin^+^ and CD133^+^ stem cells, which condition angiogenesis and tumor growth [6], or as more complicated structures including, beside tumor stem cells, endothelial cells, astrocytes, fibroblasts, macrophages, pericytes, non-stem tumor cells, and microglia [3] (Figure 1). GSCs have been demonstrated to occur in PVN by CD133 positivity [7] or by side population signature genes, aspartate beta-hydroxylase domain containing 2 (ASPHD2) or nuclear factor erythroid 2-like 2 (NFE2L2) or hypoxia-inducible factor 2 (HIF-2) [8]. Positivity to stemness antigens increases with malignancy [9]. C6 glioma xenografts with a high content of GSCs exhibit an increased microvessel density and an increased recruitment of bone marrow (BM)-derived endothelia progenitors [10].

**Figure 1:**
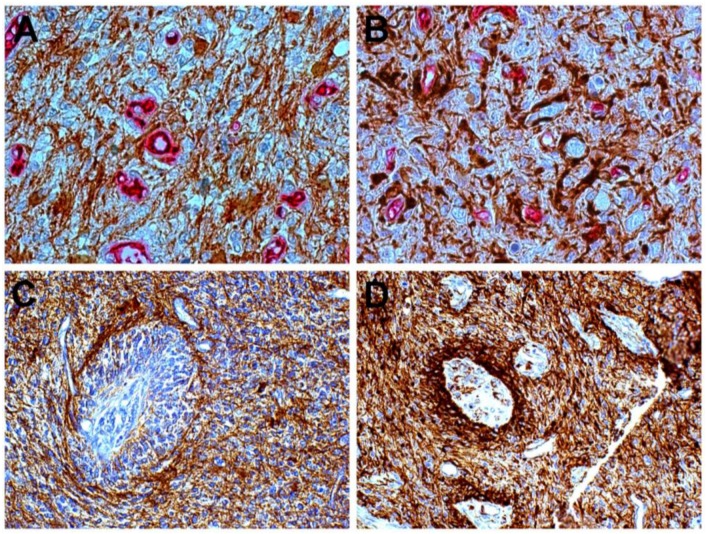
Glioblastoma. Close relationship of Nestin^+^ cells with small vessels (**A**), but not with GFAP cells (**B**); DAB, ×400; Ring of tumor cells around a vessel: the inner cells are Nestin^+^ (**C**) and GFAP^+^ cells are external (**D**), DAB, ×200.

PNN have been described to develop around circumscribed necroses where hypoxia and HIF-1/2 [4] play a central role and to contain GSCs/progenitors as in PVN [7,8]. Therefore, in both niches a fundamental feature is, therefore, the occurrence of GSCs/progenitors that condition tumor aggressiveness (growth, proliferation, migration, resistance and recurrence).

PVN are not merely repositories of stem cells [11], but they are dynamic entities that save stem cells from depletion and protect the host from their over-exuberant proliferation [12]. In GBM, vasculature is represented by simple endothelial cells, their hyperplasia or microvascular proliferations (MVP) to which not only endothelial cells, but mainly pericytes and muscle cells contribute [13,14]. The first inter-relation to be considered is the reciprocal influence between GSCs/progenitors and endothelial cells [11]. The stemness status of the former is maintained by endothelial cells *via* nitric oxide and Notch activation [3,6,15], whereas GSCs/progenitors would activate endothelial cells to proliferate, eliciting angiogenesis through vascular endothelial growth factor (VEGF) (Figure 2), to host at the tumor the BM-derived endothelial precursor cells (EPCs) promoting their differentiation into blood vessels inserted into the pre-existent vasculature [4]. In PVN, microenvironment includes the crosstalk with microglia/macrophages with their double pro-proliferation and pro-inflammatory exchanges [16,17,18,19], the function of pericytes [20,21,22,23], of reactive astrocytes, *etc.,* [24].

Hypoxia, a mechanism of primary importance in the biology and aggressive behavior of malignant gliomas [25], is fundamental in PNN. It is critically involved in the regulation of GSCs [8] of which it promotes the expansion through the phosphatidylinositol 3 kinase (PI3K)/Akt and ERK1/2 pathways; the inhibition of the latter reduces the number of GSCs [26]. The mechanism of GSC promotion still consists in Notch activation through its ligands and the final activation of target genes Hes1 and Hey1 [8,27] (Figure 2). This has been confirmed by the blockade of Notch by γ-secretase inhibition that reduces the expression of stemness antigens such as Nestin, CD133, Bmi1 and inhibits *in vitro* neurosphere formation and xenographts [28]. Hypoxia is a feature of the entire GBM, but it is particularly evident where circumscribed necroses develop and where it induces key stem cells genes such as Nanog, Oct4 and c-Myc [29]. GSCs are in fact well demonstrable not only in perinecrotic palisadings, but also in cells scattered in the proliferating tumor [7,8]. In the signaling that occurs in niches all pathways that regulate tumor progression and transformation and other processes are included, such as epidermal growth factor receptor (EGFR) gene amplification, phosphatase and tensin homolog (PTEN) mutation, PI3K/Akt, bone morphogenetic proteins (BMP), loss of heterozygosity (LOH) on critical chromosomal regions, *etc.*, as well as the intrinsic signaling such as Wnt/β-catenin, Bmi1, c-Myc, Oct4, OLIG2, Sonic Hedgehog (SHH), and Notch.

**Figure 2:**
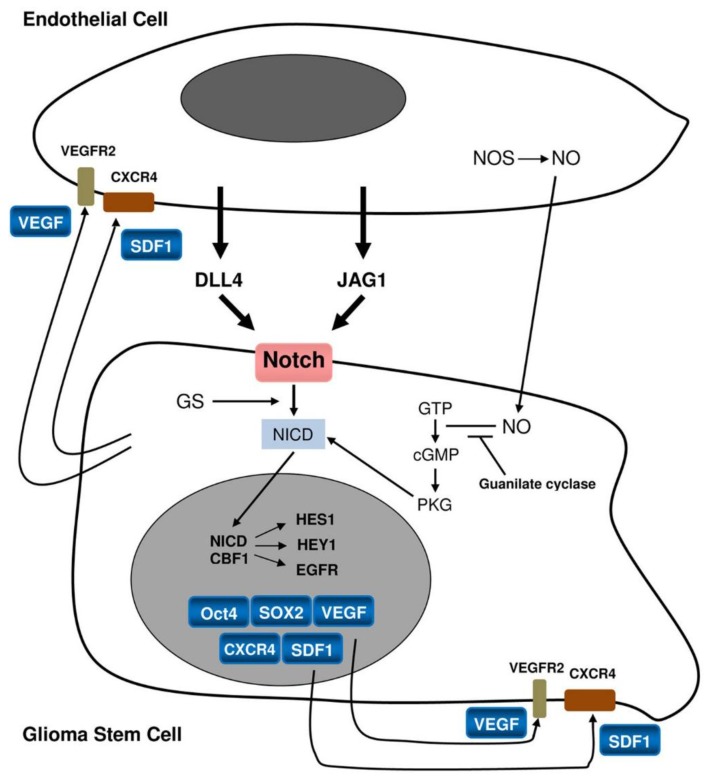
Relationship between a stem cell/progenitor and an endothelial cell.

In the discussion of the tumor microenvironment, it is mandatory to consider the supposed origin of GSCs, *i.e.*, by transformation of the normal neural stem cells (NSCs) and progenitors in relation with the so-called glioma initiating cells (GICs), the concept of which is just mentioned here. In line with the old belief that cancer cells are similar to embryonic stem cells [30], it has been established that glioma cells derive from immature glia [31,32], *i.e.*, from primitive neuroepithelial cells or NSCs [33]. GICs share properties with NSCs [34,35,36], either in the sub-ventricular zone (SVZ) or during migration. GSCs, on the other hand, share genetic alterations with gliomas [37].

Other possibilities are the origin of GSCs from oligodendroglial precursor cells (OPCs) or NG2 cells [38,39,40,41] or by dedifferentiation either from normal glia through a multistep process [42,43,44] or through dedifferentiation of tumor cells that acquire stemness properties [45] (Scheme 1). This interpretation implies that GSCs are not a cell type, but they represent a functional status [46,47,48,49] that can be acquired or lost depending on the microenvironment. Glioma heterogeneity would depend on polyclonality, *i.e.*, on genotypic and phenotypic differences acquired during proliferation, migration, also by epigenetic mechanisms [49,50] and, therefore, on the undifferentiation/differentiation status of its cell elements. The hypothesis is gaining consent that there is an equilibrium between tumor stem cells and tumor non-stem cells with the possibility of a conversion into one another regulated by the microenvironment [50]. This has been demonstrated to happen also in gliomas [1].

**Scheme 1:**
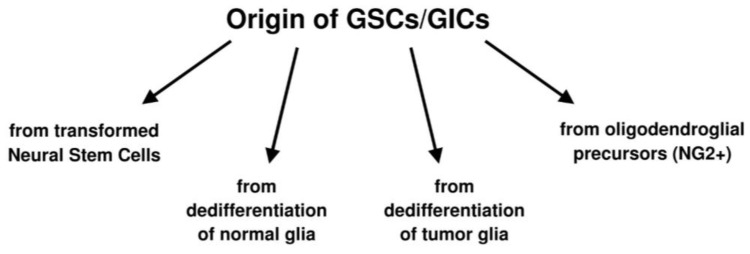
Hypotheses on the origin of glioblastoma stem cells (GSCs)/glioma initiating cells (GICs).

From the neuropathological point of view, the concept of microenvironment in gliomas materializes in its main expression sites, *i.e.*, PVN and PNN. Theoretically and conceptually, the niches have been very well-defined [3,4,5], but, practically, except for the occurrence of GSCs/progenitors, it would be very difficult to establish when and how we can recognize them in the tumor phenotype. Either the occurrence of GSCs/progenitors is mandatory for their definition or each association between vessels/necroses and tumor falls within the definition of niche. A second point of uncertainty is that the term GSCs includes progenitors in different stages of differentiation, compared to the normal cytogenesis, until they keep the quantity of stemness that allow them to proliferate, to further differentiate, to give origin to neurospheres in culture and to be tumorigenic. It is not easy to ascertain this by immunohistochemistry, unless specific antigens are used [8] or by fluorescence-activated cell sorting (FACS), even though not even by the latter a certainty can be reached [51]. One of the two: either the definition of niches in the vessels/tumor and necroses/tumor relationship is uncertain or each such relationship is a niche. It would only depend on the degree of stemness of tumor cells, including the possibility that, hypothetically, NSCs could occur in such position. This impasse could be overcome with the hypothesis of a re-programming or a conversion of tumor cells into tumor stem cells by the activity of the microenvironment [1,52]. In a series of GBMs, the possible relationship between tumor stem and non-stem cells and vessels has been analyzed and the occurrence of cells expressing stemness antigens has been described [48].

As for PNN, we showed that, at variance with the activation of GSCs by hypoxia through HIF-1/2 [4,8], the tumor stem cells/progenitors around circumscribed necroses could represent the residues of those that populated hyperproliferating areas of GBM, that occur after MRI in the enhancing area around central necrosis in which necroses develop, regulated by the microenvironment [47]. Incidentally, this subject correlates with the relationship between tumor zone composition and heterogeneity with the extent of surgical removal and outcome [53]. Circumscribed necroses have been interpreted as due to a vessel pathology with consequent ischemia/hypoxia and activation of HIF-1/2 [54,55,56]. Another hypothesis has been put forward, *i.e.*, that necrosis is due to the imbalance between the high proliferation rate of hyperproliferating areas of the tumor and the low one of endothelial cells [57]. Cell death is due either to necrosis or to apoptosis that occurs prevailingly in the palisading. The highly proliferating areas are populated by GSCs/progenitors deriving from dedifferentiated tumor cells that acquired stemness properties and that disappear after necrosis development, remaining to line the pseudopalisading as the cells spared by necrosis [1] (Figure 3 and Figure 4).

In conclusion, microenvironment by genetic and epigenetic mechanisms regulates the equilibrium between tumor stem cells and tumor non-stem cells so that the occurrence of GSCs would not imply the existence of a special type of cells, but it would be the consequence of an interplay that takes place in the microenvironment (Scheme 2). This would have therapeutic consequences, since the therapies directed to annihilate a fixed target such as GSCs as the responsible for growth, resistance and recurrence of the tumor should be converted into therapies aimed at molecular modifications of the microenvironment.

**Figure 3:**
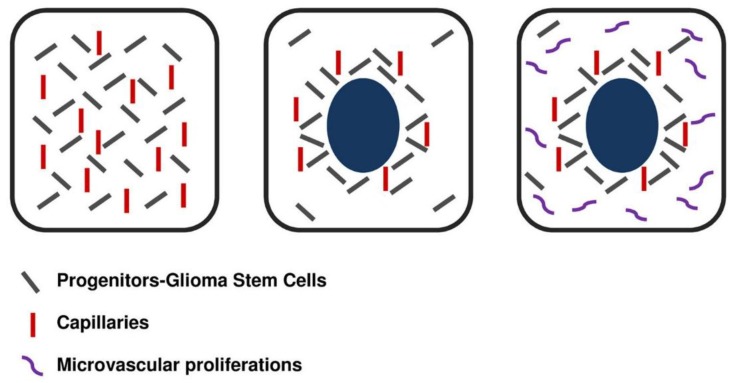
Development of necrosis in a hyperproliferating area from ischemia due to the imbalance between proliferation rate of tumor and endothelial cells.

**Figure 4:**
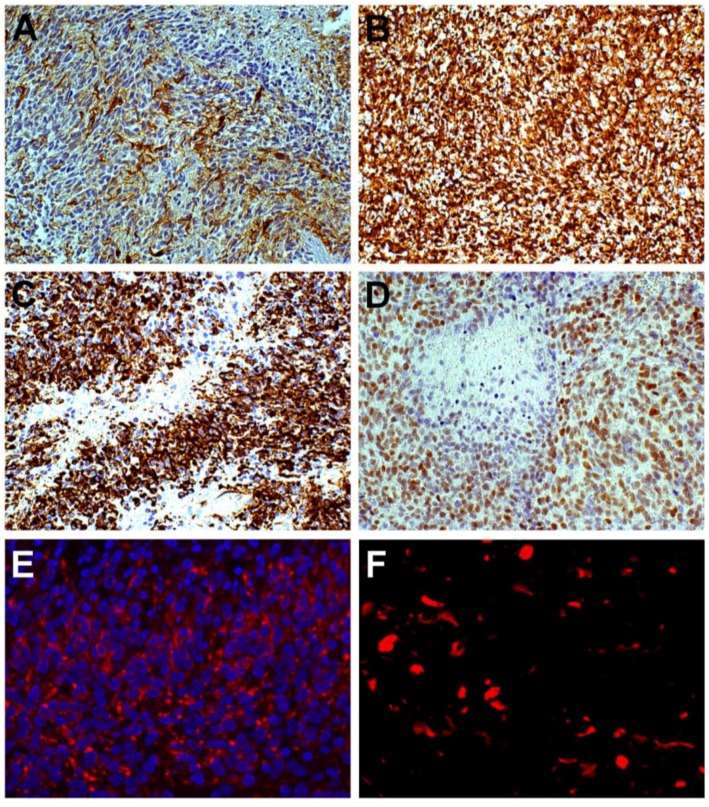
Glioblastoma. (**A**) Hyperproliferating area with scarce GFAP-positive cells, DAB, ×200; (**B**) *Id.* with abundant Nestin^+^ cells, DAB, ×200; (**C**) Circumscribed necrosis developing in a Nestin-rich hyperproliferating area, DAB, ×200; (**D**) Circumscribed necrosis developed in a SOX2-rich hyperproliferating area, DAB, ×200; (**E**) Most cells are Nestin+ in a perinecrotic palisade, immunofluorescence, ×400; (**F**) Nestin^+^ cells around a circumscribed necrosis, immunofluorescence, ×400.

**Scheme 2:**
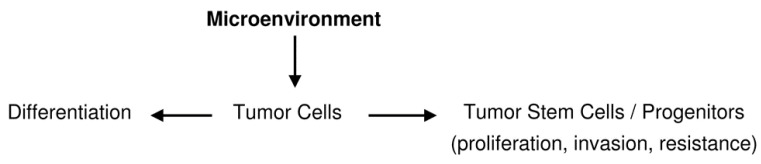
Possible dynamics of stemness and differentiation.
